# Random Practice Enhances Retention and Spatial Transfer in Force Field Adaptation

**DOI:** 10.3389/fnhum.2022.816197

**Published:** 2022-05-04

**Authors:** Michael Herzog, Anne Focke, Philipp Maurus, Benjamin Thürer, Thorsten Stein

**Affiliations:** ^1^BioMotion Center, Karlsruhe Institute of Technology, Karlsruhe, Germany; ^2^Faculty of Kinesiology, University of Calgary, Calgary, AB, Canada

**Keywords:** motor adaptation, contextual-interference effect, state-space model (SSM), spatial generalization, motor retention, variability of practice, sensorimotor learning, reaching movements

## Abstract

The contextual-interference effect is a frequently examined phenomenon in motor skill learning but has not been extensively investigated in motor adaptation. Here, we first tested experimentally if the contextual-interference effect is detectable in force field adaptation regarding retention and spatial transfer, and then fitted state-space models to the data to relate the findings to the “forgetting-and-reconstruction hypothesis”. Thirty-two participants were divided into two groups with either a random or a blocked practice schedule. They practiced reaching to four targets and were tested 10 min and 24 h afterward for motor retention and spatial transfer on an interpolation and an extrapolation target, and on targets which were shifted 10 cm away. The adaptation progress was participant-specifically fitted with 4-slow-1-fast state-space models accounting for generalization and set breaks. The blocked group adapted faster (*p* = 0.007) but did not reach a better adaptation at practice end. We found better retention (10 min), interpolation transfer (10 min), and transfer to shifted targets (10 min and 24 h) for the random group (each *p* < 0.05). However, no differences were found for retention or for the interpolation target after 24 h. Neither group showed transfer to the extrapolation target. The extended state-space model could replicate the behavioral results with some exceptions. The study shows that the contextual-interference effect is partially detectable in practice, short-term retention, and spatial transfer in force field adaptation; and that state-space models provide explanatory descriptions for the contextual-interference effect in force field adaptation.

## Introduction

Motor skills enable people to interact with the environment in many different ways. Motor skills are not innate but learned throughout life, which indicates the importance of understanding motor learning processes. In the literature, two types of motor learning are usually distinguished: (1) skill learning, which “is a set of processes associated with practice or experience leading to relatively permanent changes in the capability for skilled movement” (Schmidt et al., 2019); and (2) motor adaptation, where the motor system responds to changes in the body and/or the environment to return to a previous level of performance under these new environmental conditions (Krakauer and Mazzoni, 2011). For both types of learning, practice is the most important factor and a central question of research in motor learning is to understand how different practice protocols (e.g., amount of practice, distribution of practice or variability of practice) affect motor learning processes on different time scales.

In this regard, the contextual-interference effect (CIE) is a well-studied phenomenon in motor skill learning. The CIE states that interleaved (high contextual interference) as opposed to repetitive (low contextual interference) practice results in lower performance gains during practice but superior retention and transfer (Shea and Morgan, 1979). Originally formulated by Battig (1972) for verbal learning, a large body of research has supported the CIE in motor skill learning, especially for simple laboratory tasks but also in more complex sport tasks (for an overview; see Schmidt et al., 2019). However, compared to skill learning, the CIE has not been widely studied in the context of motor adaptation (Thürer et al., 2019). Accordingly, this study focuses on the analysis of the CIE in a motor adaptation task.

There are different experimental paradigms to analyze motor adaptation (Krakauer et al., 2019). In this study, we use the force field paradigm (Shadmehr and Mussa-Ivaldi, 1994; Shadmehr, 2017) to study the CIE in motor adaptation. Here, participants perform reaching movements and experience forces on their hand, leading them to laterally deviate from straight trajectories. The deviations predominantly result from a sensory prediction error, i.e., a mismatch between the predicted and the experienced movement (Krakauer and Mazzoni, 2011). This error is assumed to drive trial-by-trial adjustments of an internal model (Kawato, 1999; Donchin et al., 2003; Shadmehr et al., 2010; Albert and Shadmehr, 2016). Thereby, the motor commands are updated. This enables the participants to counteract successively better the perturbances and to ultimately perform a straight trajectory. This means that the participants returned to a previous level of performance (Shadmehr et al., 2010). The acquired internal model can be interpreted as a motor memory that is partially transferrable to new situations (Shadmehr, 2017). For example, there is evidence for transfer to different movement speeds and amplitudes (Goodbody and Wolpert, 1998; Joiner et al., 2010; Mattar and Ostry, 2010). Also, contralateral transfers could be shown (Criscimagna-Hemminger et al., 2003; Joiner et al., 2013; Stockinger et al., 2015). A host of literature found spatial transfer capabilities in force field adaptation, such as to different reaching directions or to arm configurations that are shifted by several centimeters (Shadmehr and Mussa-Ivaldi, 1994; Gandolfo et al., 1996; Ghez et al., 1999; Shadmehr and Moussavi, 2000; Rezazadeh and Berniker, 2019).

The adaptation progress itself resembles an exponential function with a fast initial increase followed by a slower, more gradual increase (Krakauer et al., 2019). This progress can be modeled well with linear, time-invariant (multi-) state-space models (SSMs) (Smith et al., 2006). Thereby, the error serves as input, the update of the internal model as a hidden variable, and the adjusted, subsequent movement as output (Krakauer and Mazzoni, 2011). In particular, the fast initial increase is attributed to a fast process with a high learning rate and rapid decay, and the subsequent phase to a process with a slower learning rate but greater retention (Smith et al., 2006). SSMs have successfully characterized and predicted numerous phenomena in force field adaptation (Kim et al., 2021). Thus, they offer the possibility of investigating potential processes underlying practice related to behavioral changes (Smith et al., 2006).

As described above, adaptation progress, retention, and spatial transfer characteristics in force field adaptation have been thoroughly examined. However, no study so far has explicitly investigated CIE, i.e., the different effects of interleaved and repetitive practice schedules on retention and spatial transfer in force field adaptation. Further, despite the large host of studies in motor skill learning, there is no sole hypothesis to fully explain the CIE (Wright and Kim, 2019). The three prevailing hypotheses are “elaboration-and-distinctiveness” (Shea and Morgan, 1979), “retroactive inhibition” (Shea and Titzer, 1993), and “forgetting-and-reconstruction” (Lee and Magill, 1983; Lee et al., 1985). According to the first, interleaved practice requires performing comparative and distinctive analyses on a trial-by-trial basis, which increases the cognitive effort compared to repetitive practice. This increased effort slows down the acquisition, but fosters better retention performance by a more distinct or better representation of the task in the memory. The retroactive inhibition hypothesis explains the CIE such that learning a similar task in a repetitive manner inhibits recalling a memory of a preceding, different task. However, this hypothesis is probably not valid for the CIE in motor adaptation tasks (Thürer et al., 2018, 2019). The forgetting-and-reconstruction hypothesis proposes that the action plan for a task is forgotten over time and vanishes from short-term memory. If it is repeatedly needed during random practice, it must always be reconstructed. This, in turn, slows down acquisition, but fosters retention and transfer. Due to the interplay of learning and decay, SSMs in particular enable the study of the CIE in terms of the forgetting-and-reconstruction hypothesis (Schweighofer et al., 2011).

Accordingly, this study follows a combined approach to investigate the CIE in force field adaptation. The first objective is to experimentally investigate if there is a CIE regarding retention and spatial transfer in a force field adaptation task. The second objective is to fit an extended SSM to the experimental data to infer possible latent mechanisms. We hypothesize that: (1) participants with an interleaved practice schedule achieve a lower adaptation level at practice end than participants with a repetitive schedule and adapt slower; (2) participants of the interleaved group demonstrate better retention and (3) spatial transfer; and (4) the superior effect of the interleaved practice schedule can be explained by the two-rate characteristic of the learning process.

## Materials and Methods

### Participants

Thirty-two right-handed (Oldfield, 1971), healthy female and male volunteers (age 24 ± 3 years) participated in the study. All participants were naïve to force field adaptation experiments, informed about the experimental protocol, and gave their written informed consent. The study protocol was submitted to and approved by the Ethics Committee of the Karlsruhe Institute of Technology.

### Apparatus and Task

**FIGURE 1:**
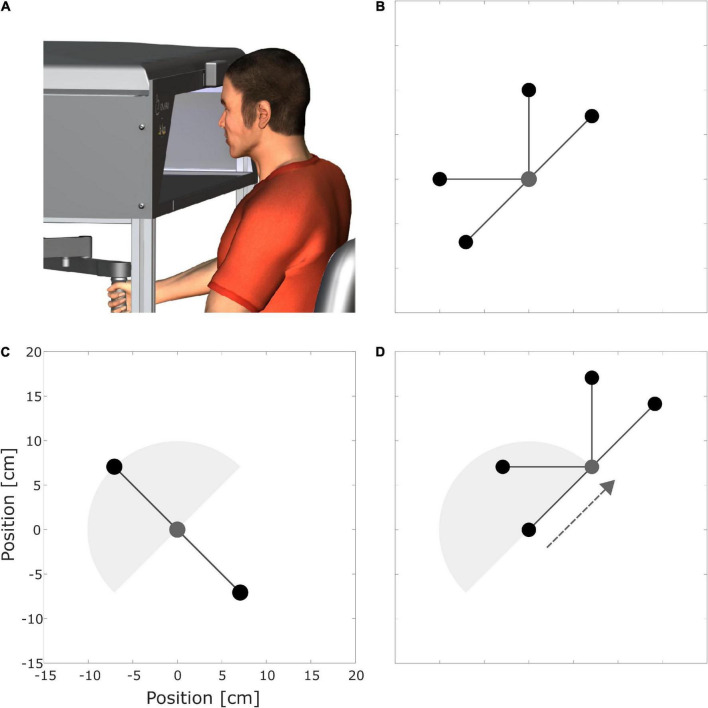
**(A)** Participant sits at a KINARM End-Point Lab (BKIN Technologies Ltd., dba Kinarm, Kingston, Canada). **(B)** Start point (0,0) in gray and target points (practice targets) in black. **(C)** Start (0,0) in gray, interpolation target (10.30 h), and extrapolation target (4.30 h) in black. **(D)** Targets with shifted origin. The dotted gray arrow illustrates the translational shift. In **(C,D)** the gray semicircle illustrates the area spanned by the practice targets, but this was not visible during the experiments.

The participants sat at a KINARM End-Point Lab (BKIN Technologies, Kingston, Canada), and performed 10 cm point-to-point movements with their right hand in the transverse plane. The manipulandum was equipped with a virtual reality display showing the handle’s position and the start and target points, but occluding vision of the handle itself, their arms, and hands (Figure 1A). Participants were instructed to reach from the start to the target within 500 ± 50 ms. When the target was reached, its color changed, providing the participants with feedback on whether the specified time was met. Shortly after the target was reached and the cursor had resided in it for 800 ms, the manipulandum moved the handle back to the start for the next trial.

To comprehensively investigate the effects of different practice schedules on spatial transfer, we considered three different spatial transfer tasks, for which literature has shown different amounts of transfer. Following the dial of a clock for orientation, the points were located as follows (Figures 1B–D): the first start point was at (0,0). The “practice targets” were positioned at 1.30, 12, 9, and 7.30 h (Figure 1B). Figure 1C shows the “interpolation” (10.30 h) and “extrapolation” (4.30 h) targets. The second starting point was shifted 10 cm to 1.30 h (Figure 1D). The remaining four targets had the same directions as the practice targets but were shifted like the second starting point (Figure 1D). We expected a good transfer for the interpolation target, as its direction is similar to the one practiced (Gandolfo et al., 1996; Castro et al., 2011; Rezazadeh and Berniker, 2019). In contrast, we expected no transfer for the extrapolation target as its direction deviates at least 90 degrees from the practice targets (Ghez et al., 1999; Castro et al., 2011). Based on studies by Ghez et al. (1999) and Shadmehr and Moussavi (2000), we expected fractional transfer for the shifted origin targets.

### Experimental Design

#### Trial Conditions

Three different trial types were used: null field (NF), force field (FF), and error clamp (EC). During NF trials, the handle was freely movable without perturbing forces. FF trials were carried out in a viscous (velocity-dependent), counter-clockwise force field. Hereby, the force field was specified by the formula


$$F=k*[cos\theta ,-sin\theta ;\;sin\theta ,cos\theta ]*[\dot{x};\dot{y}];$$

where k denotes the force field magnitude and was fixed at 20 Ns/m. The angle θ was set to 90°. The velocity components of the handle are given by the vector [ẋ; ẏ]. Accordingly, the force field always deviated the handle’s movement orthogonally to its movement direction. For EC trials, the manipulandum restricted the movement to a small channel connecting the start and end points (Scheidt et al., 2000; Joiner and Smith, 2008). Therefore, the manipulandum created virtual walls, with a wall viscosity of 10 kNs/m and a wall stiffness of 1 kN/m.

#### Group Assignment and Schedule

Thirty-two participants were randomly assigned to two groups called “blocked” and “random” (each *N* = 16, with 8 females and 8 males). The experiment consisted of five different phases: familiarization, baseline, practice, short-term test, and long-term test. The first three phases were separated by 5 min breaks, and there was a 10 min break between practice and the short-term test. The long-term test followed 24 h later. During the practice phase, there were 30 s breaks after every 80 trials, during which participants could let go of the handle but remained seated. The various phases differed in the types of trials and targets used (Table 1).

**TABLE 1:** Practice schedule.

	Familiarization	Baseline	Practice	Short-term test^§^	Long-term test^§^
Targets	pr	pr, in, ex, so	pr	pr, in, ex, so	pr, in, ex, so
Number and type of trials	120 NF	18 NF, 10 EC	720 FF, 80 EC	20 EC	10 EC, 10 FF
Trials per target	30	Practice targets: 3 NF, 1 EC Others: 1 NF, 1 EC	200	2	1 EC, 1 FF
Trial ordering *Blocked group*	Random^†^	3x pr, in, ex, so (NF), pr, in, ex, so (EC)	*N* = 4: 1.30, 12, 9, 7.30 h* *N* = 4: 12, 9, 7.30, 1.30 h* *N* = 4: 9, 7.30, 1.30, 12 h* *N* = 4: 7.30, 1.30, 12, 9 h*	*N* = 8: pr, in, ex, so *N* = 8: pr, ex, in, so	*N* = 8: pr, in, ex, so^‡^ *N* = 8: pr, ex, in, so^‡^
Trial ordering *Random group*	Random^†^	3x pr, in, ex, so (NF), pr, in, ex, so (EC)	Random^†^	*N* = 8: pr, in, ex, so *N* = 8: pr, ex, in, so	*N* = 8: pr, in, ex, so^‡^ *N* = 8: pr, ex, in, so^‡^

There was a 10 min break between practice and the short-term test, and 24 h between the short- and long-term tests. Between all other phases, there were 5 min breaks. Targets are abbreviated: practice targets (pr), interpolation (in), extrapolation (ex), and shifted origin (so). *Each target was reached 200 times before switching to the next.

*^†^The order was random, but all targets were reached within four trials. ^‡^Each target group was reached in the EC condition first and then in FF. ^§^Detailed ordering for every participant is listed in Supplementary Table 1.*

To enable the participants to become accustomed to the manipulandum and the desired movement speed, the familiarization consisted of 120 NF trials. Targets appeared in block-randomized order (4 targets × 30 blocks). The baseline phase consisted of three reaches to each of the practice targets, and one reach to each of the interpolation, extrapolation, and shifted origin targets in the NF condition. Then, all targets were approached once in the EC condition.

Practice consisted of 800 trials. The practice targets of the random group appeared in a random order, although each target was reached once within a block of four trials (interleaved practice). In contrast, each participant in the blocked group practiced one of the four practice targets 200 times before proceeding to the next (repetitive practice). Eighty EC trials were randomly interspersed. Both groups were divided into four sub-groups (*N* = 4 each), each of which began with a different practice target.

The short-term test consisted only of EC trials. Thereby, we assessed short-term retention and short-term spatial transfer. First, two blocks of practice targets appeared (retention test), followed by transfer tests. Two blocks with the inter- and extrapolation target appeared, then two blocks with the shifted-origin targets. The targets’ ordering varied within the test blocks for each participant, with one of each group (blocked and random) with the same ordering. To exclude a potentially occurring retroactive inhibition effect, the four subgroups were further divided into two sub-subgroups. The order of the targets in the retention test was equal to the order of the subgroup for the first sub-subgroup and in reverse order for the second sub-subgroup (e.g., blocked subgroup B1: 200 × 1.30 h, 200 × 12 h, 200 × 9 h, and 200 × 7.30 h; sub-subgroup 1: 1.30, 12, 9, and 7.30 h; sub-subgroup 2: 7.30, 9, 12, and 1.30 h). The exact ordering for all participants is shown in Supplementary Table 1. The long-term tests followed a similar protocol as the short-term tests, but every second reach to a target was a FF trial, viz., not an EC trial. The exact ordering is shown in Supplementary Table 1.

### Data Analysis

Kinematic data, including hand position and velocity, and forces measured at the manipulandum’s handle were recorded at 1,000 Hz with KINARM Dexterit-E software (BKIN Technologies Ltd., Kingston, ON, Canada).

#### Pre-processing

Following our previous studies (Stockinger et al., 2015), raw kinematic and force data were filtered with a fourth-order Butterworth low-pass filter and a cut-off frequency of 6 Hz (kinematic) and 10 Hz (force). Movement start and end were defined as the time points where the hand velocity exceeded or fell below 10% of the trial’s peak velocity. Segmented data were time-normalized to 101 time points using cubic spline interpolation.

#### Dependent Variables

The dependent variables were calculated with ManipAnalysis (Stockinger et al., 2012) and self-written Matlab scripts (R2020a; The MathWorks, Inc., Natick, Massachusetts, United States). Following studies by Sing et al. (2009) and Heald et al. (2018), we assessed adaptation with a kinematic and a dynamic parameter. The maximum perpendicular distance (PD_*max*_) between the participant’s trajectory and a virtual straight line connecting the start and target points served as kinematic measure on FF trials. It quantifies the net motor output as it includes all control processes involved (Stockinger et al., 2015).

While PD_*max*_ quantifies the kinematic output, the force field compensation factor (FFCF) is a dynamic measure, quantifying the participant’s force field prediction (Scheidt et al., 2000; Joiner and Smith, 2008). The FFCF was calculated on EC trials, i.e., when the kinematic error was kept to zero. The force the participant applied orthogonally toward the virtual wall was computed (F_*actual*_). The ideal force field profile F_*ideal*_ was calculated as a product of the velocity profile of the trial and the force field matrix. The FFCF was then obtained using linear regression of F_*ideal*_ and F_*actual*_ according to the formula


F(t)a⁢c⁢t⁢u⁢a⁢l=a*1F(t)i⁢d⁢e⁢a⁢l+a+0e(t).

Thereby, e denotes the error that is to be minimized in least-squares sense, and a_0_ and a_1_ are the regression coefficients of the fit. The coefficient a_0_ denotes the axis intercept. The slope a_1_ serves as the FFCF. If F_*ideal*_ coincides with F_*actual*_, the FFCF is 1. If they are unrelated, the FFCF is 0. All FFCF values after the baseline are participant- and target-specific baseline-subtracted values, to ensure only the learning-induced changes in the force profile are considered (Wagner and Smith, 2008).

The PD_*max*_ progress was investigated by fitting (Matlab lsqcurvefit) the following exponential model to the PD_*max*_ curve (Davidson and Wolpert, 2004; Stockinger et al., 2015):


PD(tr)m⁢a⁢x=a+0a*1exp(-tr/τ),

where tr is the number of the trial, and τ serves as the time constant of adaptation used to compare the adaptation progress. The scalar a_0_ represents the participant’s performance learning plateau and a_1_ the gain. Considering that the blocked group successively reached to the same and the random group to varying targets, we used the following approaches to compare the progress between the two groups. Firstly, we fit the model to the participant-specific PD_*max*_ curves of the whole practice. The fitting procedure was repeated 1,000 times with random initial values for a_*i*_ and τ. The fits with the highest *R*^2^ were taken for further analyses. Secondly, we fit the model to participant- and target-specific PD_*max*_ curves (Krakauer et al., 2000). For the target-specific fits, we used a bootstrapping procedure as individual data were noisy, sampling 1,000 times per group with 64 (4 targets × 16 participants) randomly sampled PD_*max*_ curves with replacement and random initial values. Thirdly, we fit the exponential model to the target-specific mean PD_*max*_ curves.

#### Fitting of the Extended State-Space Model to Behavioral Data

We fitted the following extended SSM to each participant’s data:


e⁢(t)=f⁢(t)-y⁢(t)

$$y\,\left(t\right)={\text{x}}_{f}\,\left(t\right)\,\text{c}\,\left(t\right)+{\text{x}}_{s}\,\left(t\right)\,\text{c}\,\left(t\right)$$

$${\text{x}}_{f}\,\left(t+1\right)={\text{A}}_{f}\,{\text{x}}_{f}\,\left(t\right)+\text{c}\,\left(t\right)\,{\text{b}}_{f}\,e\,\left(t\right)$$

$${\text{x}}_{s}\,\left(t+1\right)={\text{A}}_{s}\,{\text{x}}_{s}\,\left(t\right)+\text{c}\,\left(t\right)\,{\text{b}}_{s}\,e\,\left(t\right)$$

Our extended SSM entails a fast process **x_f_** and a slow process **x_s_** running in parallel (Smith et al., 2006; Lee and Schweighofer, 2009). Their sum produces the model output y for each trial t. Model output y and perturbation f correspond to the FFCF and their difference constitutes the error e. As it is corresponding to the FFCF, the perturbation f is always equal to one (Trewartha et al., 2014). However, because no error is experienced during a block of EC trials, the error e is set to zero during short-term retention (Albert and Shadmehr, 2018). Each process’s progress depends on the preceding error, a process-specific error-sensitive learning (b_*f*_, b_*s*_) and decay (A_*f*_, A_*s*_) rate. In the formula above, **A**_*f*_ (**A**_*s*_) is a 4 × 4 matrix with A_*f*_ (A_*s*_) value on the diagonal and zeros otherwise, **b**_*f*_ is a vector [b_*f*_ b_*f*_ b_*f*_ b_*f*_], and **b**_*s*_ analogous.

The two-rate SSM as proposed by Smith et al. (2006) cannot account for multiple targets (Schweighofer et al., 2011; Tanaka et al., 2012), as long as they cannot be averaged out over a few trials (Tanaka et al., 2012; Albert and Shadmehr, 2018). Therefore, we extended the SSMs to have them account for multiple targets (Schweighofer et al., 2011; Tanaka et al., 2012). The vector **c(t)** defines the currently active context (target direction), according to Lee and Schweighofer (2009). It contains four elements, each representing one of the practice targets if four contexts are assumed or a single one if a single context is assumed (see below).

Literature (Donchin et al., 2003; Howard and Franklin, 2015; Rezazadeh and Berniker, 2019) suggests a Gaussian-tuned trial-by-trial generalization with the mean at about the target direction, a standard deviation of about 45° and almost no transfer to ± 90°. Accordingly, the value c(t,a) for the currently active context a is 1. The values for the others c(t,b) (b ∈ {practice targets\a}) correspond to the value of the tuning function (Ingram et al., 2011).


$$c\left(t,b\right)=\frac{\Delta \left(𝒩\left(180\right),𝒩\left(b\right)\right)}{\Delta \left(𝒩\left(0\right),𝒩\left(180\right)\right)},$$

$$with𝒩\left(b\right)=\frac{1}{\sqrt{2\pi^{2}}}exp\left(-\frac{{\left(\theta \left(b\right)\right)}^{2}}{2\sigma^{2}}\right)$$

Hereby, θ(b) is the absolute angular difference between the direction of target a and the direction of target b. As only the four practice targets are learned during practice, the transfer targets’ performances are constituted at the time they appear as follows. The value c for the interpolation target is set as the sum of the average states of the fast and slow processes. The value c for the extrapolation target is set to zero as we do not expect transfer to it (Ghez et al., 1999; Castro et al., 2011). The value c for a shifted origin trial is set as if it were the practice target with the same direction. This is a simplification as transfer to shifted workspaces is evident, however, setting a specific transfer coefficient was avoided due to controversial results (Shadmehr and Mussa-Ivaldi, 1994; Shadmehr and Moussavi, 2000; Malfait et al., 2002; Criscimagna-Hemminger et al., 2003; Mattar and Ostry, 2007; Berniker et al., 2014).

Based on previous findings that prolonged intervals between trials led to considerable decrease of previously achieved adaptation levels (Huang and Shadmehr, 2007; Ethier et al., 2008; Kim et al., 2015b), we extended our SSM so it accounted for the forgetting between extended inter-trial pauses and set-breaks (Kim et al., 2015b; Albert and Shadmehr, 2018). Thus, when a set-break occurred, a factor d was used, which accounted for additional decay during breaks (Albert and Shadmehr, 2018).


A={AAd+1;b={bAdbnosetbreaksetbreak

The factor d was set at 2, 20, and 2,580 for the 30 s, 10 min and 24 h breaks, respectively, being multiples of the average inter-trial interval (Albert and Shadmehr, 2018; Coltman et al., 2019).

Fitting was performed to minimize the root mean squared error (*RMSE*) between the model output and the experimental data (Matlab fmincon). The stability of the model fits and sensitivity of the constraints and initial values were evaluated with a grid search and bootstrapping procedure (Tanaka et al., 2012; McDougle et al., 2015; Sadeghi et al., 2018). Bootstrapping was performed 1,000 times per group with 16 randomly sampled participants with replacement and random initial values. We varied the number of processes (4-slow-1-fast, 1-slow-4-fast, and 4-slow-4-fast). We excluded 1-fast-1-slow as such a model would only be able to account for performance decreases after target changes by altering the decay parameter d or an overall worse fit. In case of the 4-slow-1-fast model, A_*f*_ and b_*f*_ were scalar values. Analogously, for the 1-slow-4-fast model, A_*s*_ and b_*s*_ were scalar values. We varied the search space for A_*f*_ ∈ {]0,1[,]0.5,1[,]0.5,0.9[}, A_*s*_ ∈ {]0,1[,]0.9,1[}, b_*f*_ ∈ {]0,1[,]0,0.5[}, and b_*s*_ ∈ {]0,1[,]0,0.5[} (Albert and Shadmehr, 2018; Forano and Franklin, 2020). Fitting was robust with respect to the constraints (Supplementary Table 2), so we chose a 4-slow-1-fast model as it can reproduce a larger amount of force field adaptation phenomena (Lee and Schweighofer, 2009). Parameters were constrained to 0.5 < A_*f*_ < 0.9 < A_*s*_ < 1 and 0 < b_*s*_ < b_*f*_ ≤ 0.5, ensuring each process met the appropriate scale (McDougle et al., 2015; Forano and Franklin, 2020). Initial values of x_*f*_ and x_*s*_ were constrained to be within [0,0.5], as no participant showed an initial FFCF value > 0.5.

### Statistics

#### Adaptation Progress

Adaptation to the force field during the practice phase was assessed with ANOVAs (Group: Blocked vs. Random, Time: Start, End) on the two dependent variables PD_*max*_ and FFCF. For PD_*max*_, the first eight and last eight trials of the practice phase were used for both groups. This number of trials was selected so that each target direction was included twice in each sample. For FFCF, each participant’s first and last EC trial was selected to constitute the start and end sample. The PD_*max*_ progresses were compared between the groups with a Mann-Whitney *U*-test on the time constant of adaptation τ.

#### Retention

To test for differences between groups, short-term retention was tested with one ANOVA on FFCF values (Group: Blocked vs. Random, Time: Practice end, Short-term) and long-term retention with two separate ANOVAs on PD_*max*_ and FFCF values (Group: Blocked vs. Random, Time: Practice end, Long-term). The sample for “practice end” constituted the last four trials of the practice (blocked) or the last trials per target (random). The first four EC trials of the short-term retention and the four EC trials (FF for PD_*max*_) of the long-term retention were selected, respectively, for the short-term and long-term sample. For each time point, values were averaged per participant, so that each sample contained 16 values per group.

#### Spatial Transfer

Spatial transfer was tested in two steps. First, we determined whether transfers had taken place with one-sample *t*-tests vs. 0 separately for each group and spatial transfer task. Second, if there was transfer, we tested for differences between the groups with *t*-tests as we expected differences in the amount of transfer between the groups. These tests were repeated for short-term (FFCF values) and long-term (PD_*max*_ and FFCF values) tests. All short-term samples consisted of participant-specific mean values. All long-term samples consisted of single-trial values.

#### Modeling Results and Robustness

To assess whether the SSM reflects behavioral findings, all tests for adaptation, retention, and spatial transfer were carried out on the predicted model data. Additionally, we tested whether the slow and fast process at the practice end, as well as the error-sensitive learning rates, differ between the groups.

The ranges of the 95% confidence intervals (CIs) were determined by the 2.5^th^ and 97.5^th^ percentile values over every 1,000 fits. For all statistics conducted, the significance level was set *a priori* at two-sided *p* = 0.05. The normal distribution of the data was tested with the Kolmogorov-Smirnov test, and homoscedasticity with Levene’s test. If several analyses were performed regarding the same construct, the Holm-Bonferroni method was used to adjust the significance level of the *post-hoc t*-tests. The effect sizes were determined with partial eta squared (η*_*p*_*^2^), Cohen’s *|d|* or Cohen’s *|r|* (Mann-Whitney *U*-test). Mean and standard deviation of *R*^2^ were calculated with forth-and-back Fishers z-transformations. All statistical tests were carried out in SPSS (IBM Corp., v26.0. Armonk, NY).

## Results

### Practice Performance

**FIGURE 2:**
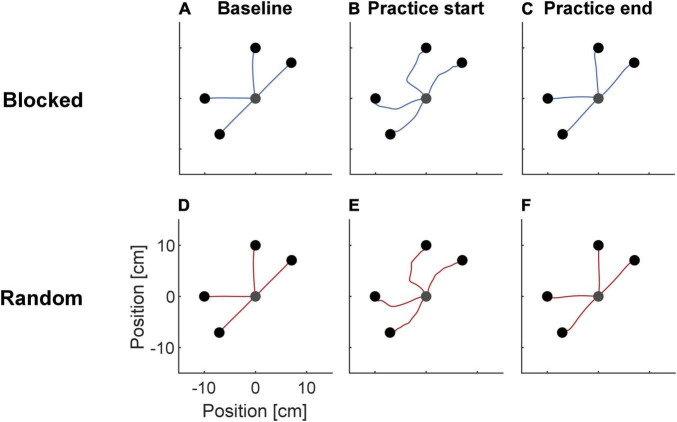
Mean trajectories of the NF baseline **(A,D)**, the first **(B,E)** and last FF practice trials **(C,F)** separated for the two groups. At practice start, trajectories deviated from the straight trajectories seen during baseline, but became similar to baseline again at practice end.

Participants’ hand trajectories in both groups during baseline, start, and end of practice resembled those typically observed in force field adaptation (Figure 2; Shadmehr and Mussa-Ivaldi, 1994). During baseline, trajectories were almost straight. At practice start, they showed high deviations along the force field direction. At the end of practice, the trajectories resembled those of the baseline phase.

**FIGURE 3:**
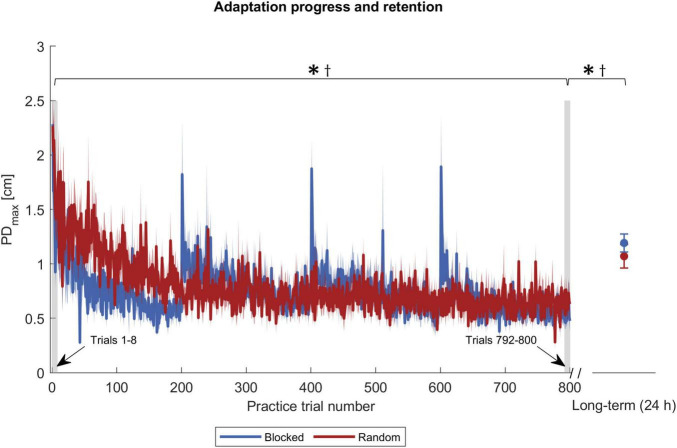
Adaptation progress and retention by PD_*max*_. The blue and red solid curves show the mean values of the respective groups, and the shaded area the corresponding standard error. The gray shaded rectangles pinpoint the trials used for statistics. Symbols indicate statistically significant differences (*p* < 0.05) in adaptation level for the blocked group (*) and the random group (†) obtained by *post-hoc t*-tests.

**FIGURE 4:**
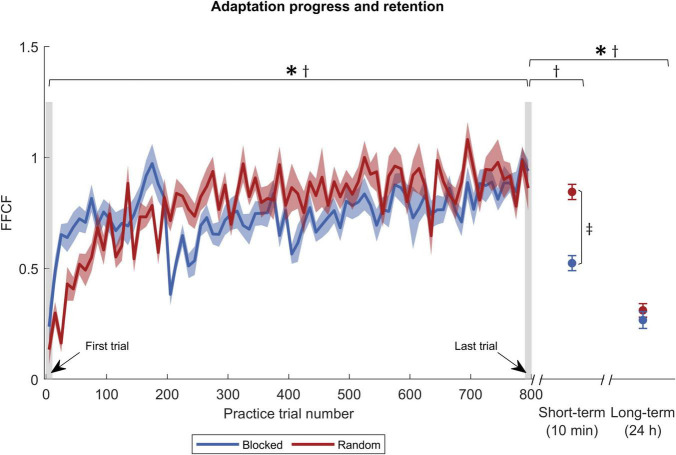
Adaptation progress and retention by FFCF. The blue solid curve shows the FFCF values of the blocked group, and red of the random group. The gray shaded rectangles pinpoint the trials used for statistics. Symbols indicate statistically significant differences (*p* < 0.05) in adaptation level for the blocked group (*), the random group (†), and between the groups (‡) obtained by *post-hoc t*-tests.

We analyzed adaptation with the two variables PD_*max*_ and FFCF. Their progression during the practice is shown in Figures 3, 4. Remarkably, FFCF (PD_*max*_) values of the blocked group showed negative (positive) peaks around trial numbers 200, 400, and 600, i.e., every time the target changed for the blocked group.

To test the first part of our first hypothesis that participants with an interleaved practice schedule achieve a lower adaptation level at practice end than participants with a repetitive schedule, we used one ANOVA for the PD_*max*_ and one for the FFCF values. We expected a significant time and interaction effect. The ANOVA with respect to the PD_*max*_ values showed a time effect [*F*_(1,30)_ = 65.431, *p <* 0.001, η*_*p*_*^2^ = 0.686], confirming that the participants adapted. The ANOVA showed no group [*F*_(1,30)_ = 1.118, *p* = 0.299, η*_*p*_*^2^ = 0.036] or interaction effect [*F*_(1,30)_ = 0.168, *p* = 0.685, η*_*p*_*^2^ = 0.006]. Using *post-hoc* tests, we compared the time effect separately for the two groups and found differences in both cases [blocked: *t*(15) = 4.197, *p <* 0.001, *|d|* = 1.049; random: *t*(15) = 10.525, *p <* 0.001, *|d|* = 2.631; Figure 3], which indicates that both groups adapted. However, there was no group difference regarding the adaptation level at practice end.

In addition, we tested the force field prediction with the FFCF. Again, we found a time effect [*F*_(1,30)_ = 104.641, *p <* 0.001, η*_*p*_*^2^ = 0.777], but neither a group [*F*_(1,30)_ = 0.775, *p* = 0.386, *η*_*p*_^2^ = 0.025] nor an interaction effect [*F*_(1,30)_ = 1.176, *p* = 0.287, η*_*p*_*^2^ = 0.038]. *Post-hoc* tests also showed a time effect for both groups [blocked: *t*(15) = −7.846, *p <* 0.001, *|d|* = 1.962; random: *t*(15) = −6.961, *p <* 0.001, *|d|* = 1.740; Figure 4]. Consequently, the FFCF yielded the same results as the PD_*max*_.

**FIGURE 5:**
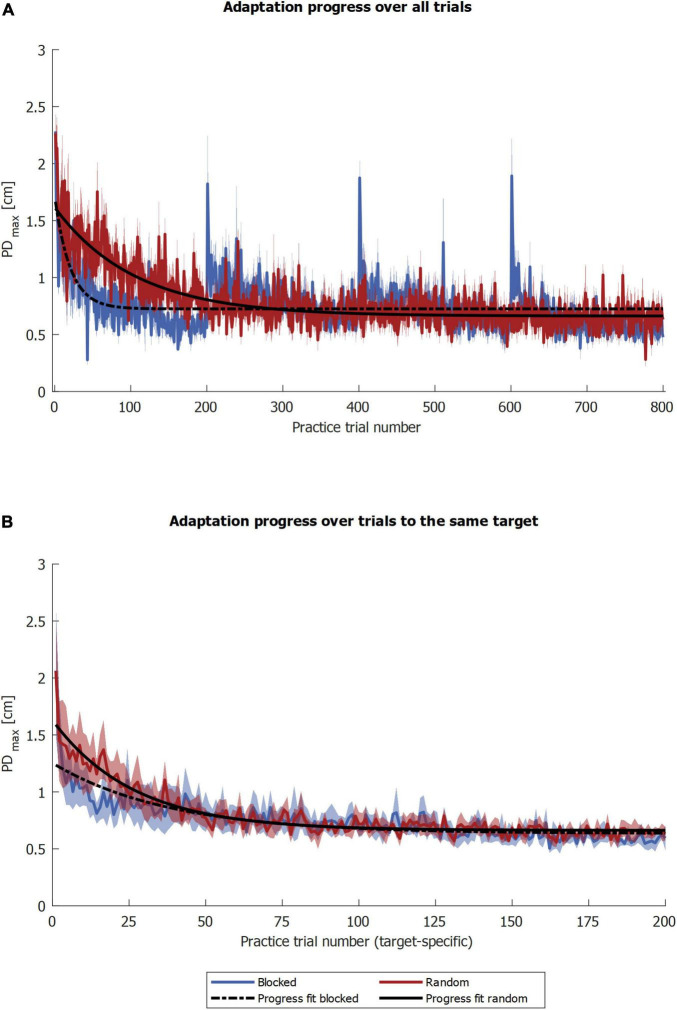
PD_*max*_ progression fits. The blue and red solid curves show the mean values of the respective groups, and the shaded area the corresponding standard error. The black curves illustrate the PD_*max*_ progression fits, the fit over the groups’ means. In **(A)** the fits were calculated over all trials and in **(B)** by target.

To test the second part of our first hypothesis that the random group adapts slower, we compared the PD_*max*_ progress. Regarding the whole curve of practice, the time constant of adaptation τ was higher for the random group, indicating slower adaptation compared to the blocked group [*U* = 57.000, *p* = 0.007; random: τ = 77 trials (SEM 10), *R*^2^ = 0.133 (SEM 0.044); blocked: τ = 22 trials (SEM 19), *R*^2^ = 0.175 (SEM 0.023), Figure 5A]. Regarding the target-specific curve of the practice, i.e., without intervening trials for the random group, the median time constant of adaptation τ was 23.6 for the random and 19.1 trials for the blocked group. However, fits were poor, as 21.4% of the whole pool of bootstrap samples yielded an *R*^2^ below 0.1, and the confidence intervals were (0.83, 327.12) for the random and (0.35, 941.9) trials for the blocked group. As a third step, we compared the two groups based on the fit to their target-specific mean progressions. Here, the time constant of adaptation τ was 37.9 for the random group and 23.9 trials for the blocked group (Figure 5B). The quality of the fit *R*^2^ was 0.75 (random group) and 0.85 (blocked group).

In summary, the statistical results of the practice phase show that both groups adapted to the force field. Compared to the blocked group, the random group did not reach a different adaptation level at practice end but adapted slower. Therefore, we cannot confirm the first part of our first hypothesis that participants with an interleaved practice schedule achieve a lower adaptation level at practice end, but can confirm that adaptation is slower in the random group.

### Retention

Our second hypothesis was that random practice improves retention. We tested for retention at two time points: 10 min (short-term retention) and 24 h (long-term retention) after practice. For each comparison, we used ANOVA to compare the adaptation levels at the end of practice to those of the retention test.

The ANOVA for the short-term retention revealed time [*F*_(1,30)_ = 11.992, *p* = 0.002, η*_*p*_*^2^ = 0.286], group [*F*_(1,30)_ = 6.317, *p* = 0.018, η*_*p*_*^2^ = 0.174], and interaction effects [*F*_(1,30)_ = 5.494, *p* = 0.026, η*_*p*_*^2^ = 0.155] for the FFCF. With *post-hoc* tests, we only found a time effect for the blocked group [*t*(15) = 7.513, *p <* 0.001, *|d|* = 0.879], which revealed that the adaptation level decreased from practice end to short-term retention test. In addition, we found that the random group showed a superior performance in the short-term retention test compared to the blocked group [*t*(30) = −5.854, *p <* 0.001, *|d|* = 2.138; Figure 4].

We used two ANOVAs to test for long-term retention, one for the PD_*max*_ values and one for FFCF values. For PD_*max*_, we found a time effect [*F*_(1,30)_ = 89.390, *p <* 0.001, η*_*p*_*^2^ = 0.749], indicating a decrease of the adaptation level. We found neither a group [*F*_(1,30)_ = 0.002, *p* = 0.962, η*_*p*_*^2^ < 0.001], nor an interaction effect [*F*_(1,30)_ = 4.182, *p* = 0.050, η*_*p*_*^2^ = 0.122]. Hence, the performance did not differ between the groups. *Post-hoc* tests revealed a time effect for both groups [blocked: *t*(15) = −8.081, *p <* 0.001, *|d|* = 2.020; random: *t*(15) = −5.273, *p <* 0.001, *|d|* = 1.318; Figure 3], indicating that both groups’ adaptation levels were lower 24 h after practice end. We also conducted an ANOVA on the FFCF values. It also revealed a time effect [*F*_(1,30)_ = 123.535, *p <* 0.001, η*_*p*_*^2^ = 0.805] but likewise no group [*F*_(1,30)_ = 0.154, *p* = 0.697, η*_*p*_*^2^ = 0.005] or interaction effect [*F*_(1,30)_ = 0.076, *p* = 0.784, *η*_*p*_^2^ = 0.003]. As with PD_*max*_, *post-hoc* tests revealed a time effect for both groups [blocked: *t*(15) = 11.299, *p <* 0.001, *|d|* = 2.825; random: *t*(15) = 6.275, *p <* 0.001, *|d|* = 1.569, Figure 4].

In summary, the random group’s retention was better 10 min after practice but did not differ from the blocked group 24 h after practice. Hence, we can accept our second hypothesis regarding short-term retention, but must reject it regarding long-term retention.

### Spatial Transfer

**FIGURE 6:**
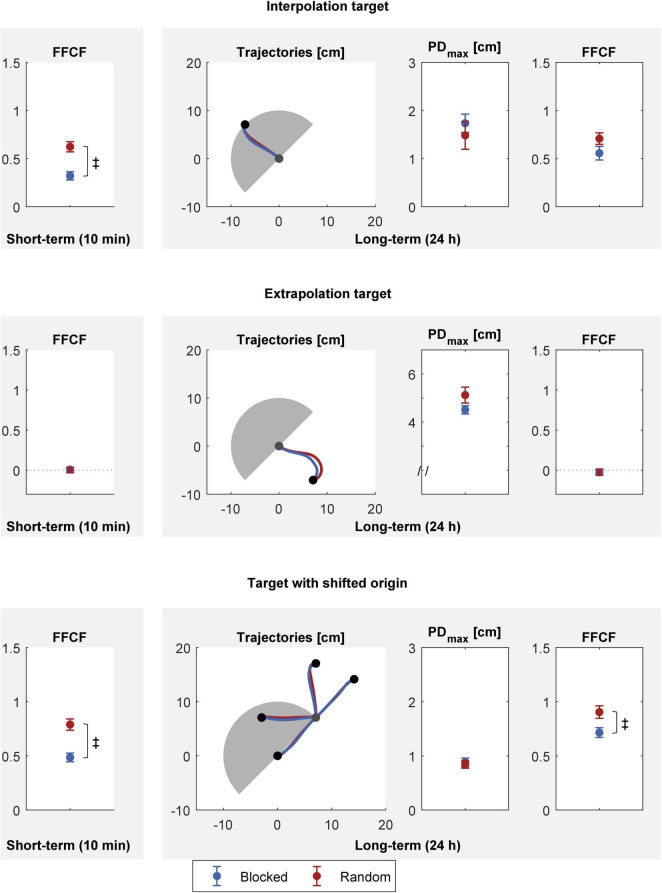
Spatial transfer 10 min (left column) and 24 h (right column) after practice. Top: interpolation target, middle: extrapolation target, bottom: targets with shifted origin. All values are mean and standard error over the blocked (blue) and random (red) groups. The gray shaded semicircle illustrates the area spanned by the practice trials for orientation. Trajectories are means over the groups. Group differences (*p* < 0.05) are indicated by the ‡ symbol.

Our third hypothesis was that random practice improves spatial transfer. We tested for transfer at two time points: 10 min (short-term transfer) and 24 h (long-term transfer) after practice, with three different kinds of targets: interpolation, extrapolation, and shifted origin (Figure 6).

For the short-term transfer, we first tested for every transfer task whether there was transfer for either group. If there was transfer, we tested which group performed better. Both groups showed transfer for the interpolation [blocked: *t*(15) = 7.490, *p <* 0.001, *|d|* = 1.934; random: *t*(15) = 11.821, *p <* 0.001, *|d|* = 3.052] and shifted origin targets [blocked: *t*(15) = 12.179, *p <* 0.001, *|d|* = 3.145; random: *t*(15) = 15.122, *p <* 0.001, *|d|* = 3.904]. No group showed transfer for the extrapolation target [blocked: *t*(15) = 0.279, *p* = 0.784, *|d|* = 0.072; random: *t*(15) = 0.036, *p* = 0.972, *|d|* = 0.009]. Then, we tested for differences between the blocked and the random group on the interpolation and shifted origin target. The random group showed a better interpolation and shifted origin transfer than the blocked group [interpolation: *t*(30) = −4.453, *p <* 0.001, *|d|* = 1.626; shifted origin: *t*(30) = −4.627, *p <* 0.001, *|d|* = 1.689].

Analogously to the short-term transfer tests, we first tested whether the groups showed long-term transfer to the different targets. Like with the short-term tests, both groups showed transfer for the interpolation [blocked: *t*(15) = 7.900, *p <* 0.001, *|d|* = 2.050; random: *t*(15) = 11.871, *p <* 0.001, *|d|* = 3.065] and shifted origin targets [blocked: *t*(15) = 15.875, *p <* 0.001, *|d|* = 4.099; random: *t*(15) = 15.685, *p <* 0.001, *|d|* = 4.043]. No group showed transfer for the extrapolation target [blocked: *t*(15) = −0.913, *p* = 0.376, *|d|* = 0.236; random: *t*(15) = −0.634, *p* = 0.535, *|d|* = 0.164]. We then examined if there was a group difference for the interpolation and shifted origin targets. The groups did not differ for the interpolation target [PD_*max*_: *t*(30) = 0.727, *p* = 0.473, *|d|* = 0.266; FFCF: *t*(30) = −1.642, *p* = 0.111, *|d|* = 0.600]. Regarding the long-term tests for the shifted origin targets, we found that PD_*max*_ values did not differ between the groups [*t*(29.726) = 0.342, *p* = 0.734, *|d|* = 0.125]. However, we found better transfer for the random group regarding the FFCF [*t*(30) = −2.582, *p* = 0.015, *|d|* = 0.943].

In summary, the random group revealed a better interpolation test performance than the blocked group 10 min after practice. We found no group difference 24 h after practice for the interpolation test. No group showed transfer to the extrapolation target, neither 10 min, nor 24 h after practice. In the shifted origin transfer task, the random group outperformed the blocked group 10 min as well as 24 h after practice (FFCF).

We hypothesized that interleaved practice fosters transfer but found mixed results. We can therefore only partially accept the hypothesis.

### State-Space Model to Model the Contextual-Interference Effect

#### General Characteristics of the Model Data

**FIGURE 7:**
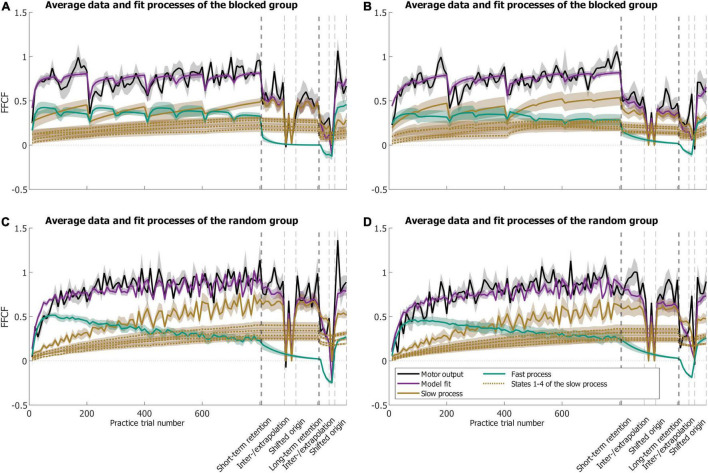
Results of the SSM fit showing mean motor output (black) and model fit (purple) with slow (brown) and fast (green) processes. The thin dotted brown lines illustrate the different states of the slow process. The dashed vertical lines separate the different phases. Means are calculated over the eight participants of each group (blocked: **A,B**; random: **C,D**) whose schedules start with the extrapolation before the interpolation target **(A,C)** or vice versa **(B,D)** during the spatial transfer phase.

The SSM captured the overall adaptation progress, retention and transfer for both groups [*RMSE_*blocked*_* = 0.27 (CI 0.03), *RMSE_*random*_* = 0.31 (CI 0.02), *R*^2^*_*blocked*_* = 0.78 (CI 0.04), *R*^2^*_*random*_* = 0.70 (CI 0.04); Figure 7]. The error-sensitive learning rates were b_*f,blocked*_ = 0.31 (CI 0.07), and b_*f,random*_ = 0.23 (CI 0.05), as well as b_*s,blocked*_ = 0.04 (CI 0.03), and b_*s,random*_ = 0.04 (CI 0.01). The decay rates were A_*f,blocked*_ = 0.79 (CI 0.10), and A_*f,random*_ = 0.86 (CI 0.05), as well as A_*s,blocked*_ = 0.99 (CI 0.01), and A_*s,random*_ = 0.99 (CI 0.01). The decay factors during breaks were d_*blocked*_ = 1.05 (CI 0.56), and d_*random*_ = 0.23 (CI 0.21). Differences in the learning rates of the fast process between the groups were not significant but revealed a large effect size, indicating a slower rate and thus a slower adaptation for the random group [b_*f,blocked*_ vs. b_*f,random*_: *t*(26.33) = −2.052, *p* = 0.050, *|d|* = 0.800].

We tested for differences in the processes’ values at the end of practice as they can possibly explain differences in the retention performances. For the slow process, the difference was not significant with a medium effect size [blocked: mean 0.51 (SE 0.05); random: mean 0.66 (SE 0.06), *t*(30) = 1.802, *p* = 0.082, *|d|* = 0.658]. The difference of the fast process’ activity was not significant either, with a weak correlation [blocked: mean 0.30 (SE 0.04); random: mean 0.24 (SE 0.04); *U* = 96.000, *Z* = −1.206, *p* = 0.239, *|r|* = 0.22].

#### Additional Analysis of the Model Data

To examine in more detail whether the model can replicate the performance trends over time induced by the random and blocked practice protocols, the same statistics as for the FFCF values (section “Practice Performance, Retention, and Spatial Transfer) were calculated from the model data and can be found in Supplementary Material 3. The statistical results of the model data were consistent with the behavioral results except for the practice start, the extrapolation target, and the long-term retention test. Thus, with regard to our fourth hypothesis, our SSM allows to provide and discuss explanatory mechanisms underlying some, but not all behavioral results.

## Discussion

The CIE is a well-studied phenomenon in motor skill learning. It states that interleaved (high contextual interference) as opposed to repetitive (low contextual interference) practice results in lower performance gains during practice, but superior retention and transfer (Shea and Morgan, 1979; Schmidt et al., 2019). The aims of the study were to investigate whether a CIE can be observed with respect to retention and spatial transfer in a force field adaptation task, and whether a SSM can reproduce the CIE and thus partly explain the underlying mechanisms of the CIE. The main findings of our study are: (1) a random practice schedule does not lead to different performance levels at practice end but to a slower adaptation than a blocked schedule. A random schedule is superior to a blocked schedule in (2) short-term retention and (3) spatial transfer. (4) SSMs reflect the experimental findings with some exceptions and provide possible explanatory mechanisms.

### Random Practice Does Not Lead to Different Performance Levels at Practice End but to a Slower Adaptation

Participants of both groups adapted to the force field perturbation. Based on the typical results of the CIE in skill learning (Wright and Kim, 2019), we expected and hypothesized that the adaptation at practice end would be worse for the random group than for the blocked group. However, we found no difference between the groups. Former studies on the CIE in our lab are inconsistent in this regard. The finding in this study concurs well with Thürer et al. (2017, 2019) but differs to Thürer et al. (2018). In the latter, a difference was only found for the enclosed area parameter (kinematic error) but not for the FFCF. However, these studies cannot be directly compared to ours, since they varied the force field magnitude rather than the reaching direction. A possible explanation for the same adaptation level at practice end is the long duration of the practice phase of our study. Usually, adaptation progression plateaus after 300−600 trials when different reaching directions are practiced (Gandolfo et al., 1996; Shadmehr and Brashers-Krug, 1997). Other CIE studies have also shown that adverse effects of random practice can be overcome during long acquisition phases (Maslovat et al., 2004; Pauwels et al., 2014).

Participants with a random schedule adapted slower than those with a blocked schedule, as indicated by the blocked group’s lower time constant of adaptation and the higher error-sensitive learning rate of the fast process (large effect size). The difference in the higher error-sensitive learning rate of the fast process indicates that the blocked group shows faster adaptation, especially in the movements at the beginning. The finding that participants in the blocked group adapted faster is in good agreement with CIE findings in the skill learning literature (Shea and Morgan, 1979; Magill and Hall, 1990) as well as with motor adaptation tasks (Thürer et al., 2018). However, not all studies explicitly compared adaptation speed (Thürer et al., 2017, 2019). Although not explicitly measured, but apparent, the blocked group seemed to adapt faster in the study of Schweighofer et al. (2011). In motor adaptation studies, there is support that increased environmental variability slows down adaptation or, in other words, a consistent environment speeds up the adaptation rate (Wei and Körding, 2009; Gonzalez Castro et al., 2014). Nonetheless, to the best of our knowledge, no study so far has compared a blocked with a random schedule regarding different movement directions in terms of adaptation speed. With the help of the SSMs, this could be described as follows. The faster adaptation can be explained by the fact that in blocked practice, the learning gain toward the next trial is maximal, since the same movement direction is practiced. In contrast, the learning gain after a trial in random practice only partially serves the next trial, as it has a different movement direction (section “State-Space Models Provide Further Explanations for the Contextual-Interference Effect/Practice”).

During practice, the blocked group showed a decrease in the FFCF and an increase in PD_*max*_ every time the target changed. Yet, the values did not fully revert to the baseline level. A possible explanation could be the breaks that took place after 200 trials. A closer look at behavioral results of force field adaptation studies (Taubert et al., 2016; Heald et al., 2018) also showed that performance decreases after short breaks, although the performance decreases are much smaller in these studies than in our study. Also, the random group took the same breaks, but no distinct steps are visible in their performances. Therefore, we suggest, with the help of our SSM, that the performance decrease can be explained by the contextual switches when a new target appeared. Due to the constant target change, the random group learned each target equally, either as it was practiced itself or by the Gaussian trial-by-trial generalization. This resulted in constant fluctuations rather than distinct steps in the adaptation progress. In contrast, the blocked group always learned the recurrent target the most and the others only by the Gaussian trial-by-trial generalization. The target after the target change was therefore less learned by the blocked group which resulted in the visible steps (section “State-Space Models Provide Further Explanations for the Contextual-Interference Effect/Practice”).

### Random Practice Yields Better Short-Term Retention but Not Necessarily Long-Term Retention

We hypothesized that random practice results in better retention performance. Therefore, we assessed the performance 10 min (short-term) and 24 h (long-term) after practice. While short-term retention benefitted from random practice, long-term retention did not. Furthermore, for both groups, long-term retention showed a lower adaptation level than short-term retention. These results match with those of Schweighofer et al. (2011) for both short- and long-term retention. However, based on our SSM, we cannot fully support in our study that the better retention performance is merely due to a higher level of the slow process (section “State-Space Models Provide Further Explanations for the Contextual-Interference Effect/Retention”). Our results on long-term retention are partially consistent with our previous studies. Thürer et al. (2017) found a difference only in FFCF, but not in PD_*max*_. While Thürer et al. (2018) did not find a group difference, Thürer et al. (2019) did find an advantage in the random group (only kinematic metric assessed). However, these comparisons are difficult as in these studies force field magnitudes were varied rather than movement directions. A better retention performance is often seen for randomly practicing groups in motor skill learning (Shea and Morgan, 1979). It must be noted, however, that motor adaptation is just a temporary transient adjustment of an existing internal model and that, generally, adaptation rapidly returns to baseline performance, which is in stark contrast with skill learning (Krakauer et al., 2019).

Factoring out differences in the practice schedule, there is much support in the literature stating that retention worsens with time in motor adaptation (Huberdeau et al., 2015b; Krakauer et al., 2019). Thereby, the passage of time plays a crucial role as the adapted state passively decays over time without any interfering trials in-between (Criscimagna-Hemminger and Shadmehr, 2008; Kitago et al., 2013). Furthermore, the adapted state also reverts toward baseline if EC trials are inserted (Scheidt et al., 2000; Kitago et al., 2013). These two findings concur well with the results of our retention tests: long-term retention is worse than short-term retention. In between the two tests, there was a 24 h pause and the short-term retention trials were only EC trials. However, the decrease in performance after 24 h without practice can be caused by a warm-up decrement being a temporary loss of an internal state that had been acquired (Kantak and Winstein, 2012; Schmidt et al., 2019). Therefore, if we had inserted a few FF trials before the long-term retention tests, maybe a group difference would have been visible.

### Random Practice Yields Better Short-Term Transfer but Not Necessarily Long-Term Transfer

To the best of our knowledge, studies so far have only examined the influence of the CIE on intermanual but not spatial transfer in force field adaptation (Thürer et al., 2018, 2019). According to skill learning studies (Goode and Magill, 1986; Wright and Kim, 2019), we expected a superior transfer performance from the random group. In light of this, and for the purpose of a comprehensive examination of spatial transfer, we investigated three different spatial transfer tasks: interpolation, extrapolation, and shifted origin. Since the time passing between practice and test trials plays a major role in force field adaptation (Criscimagna-Hemminger and Shadmehr, 2008; Krakauer et al., 2019), first short- and then long-term transfer test results are discussed separately.

#### Short-Term

Both groups showed transfer for the interpolation and shifted origin targets. Remarkably, performance decreased to no transfer for the extrapolation target.

We then found benefits for the random group compared to the blocked group in short-term transfer for the interpolation target as well as for the shifted origin targets. Our SSMs provide a possible explanation, relating the better transfer in the random group to the higher and more balanced activity of the slow process (section “State-Space Models Provide Further Explanations for the Contextual-Interference Effect/Transfer”).

Factoring out the group differences, the superior transfer performance in the short-term interpolation task compared to the extrapolation task finds support in the literature. Many studies have shown that transfer is local to the practiced movement directions in the work space and decreases with increasing angular difference between the targets (Gandolfo et al., 1996; Castro et al., 2011; Rezazadeh and Berniker, 2019). The performance results of both groups in the inter- and extrapolation tasks correspond to these findings. In addition, we found that practicing in one workspace transfers to a shifted one. These results correspond to previous studies (Shadmehr and Mussa-Ivaldi, 1994; Shadmehr and Moussavi, 2000; Malfait et al., 2002; Criscimagna-Hemminger et al., 2003; Mattar and Ostry, 2007; Berniker et al., 2014). The shifted origin targets had the same direction as the practice targets in view of extrinsic coordinates. Yet, we did not explicitly control for the coordinate system in which the targets are moved. Furthermore, there is controversy over which coordinate systems are responsible for successful transfer (Franklin et al., 2016).

#### Long-Term

Analogously to the short-term transfer, we first separately tested both groups for transfer and then tested for differences between the groups. Both groups showed transfer for the interpolation and shifted origin trials but not for the extrapolation target. This is alike our findings for the short-term transfer. However, in the long-term transfer tests, the random group did not outperform the blocked group, except for the transfer test for the shifted origin trials. For the latter, we only saw benefits when we assessed performance with the FFCF but not with PD_*max*_. Potentially, this is due to the different control mechanism the two parameters quantify (Stockinger et al., 2015): the FFCF serves as a measure of the force field prediction and thus the internal model, whereas the PD_*max*_ reflects the net motor output. The theoretical framework of optimal feedback control (OFC) (Todorov and Jordan, 2002; Scott, 2004; Todorov, 2004) and its extension robust optimal feedback control (Crevecoeur et al., 2019) may help understand why differences in FFCF values but not in PD_*max*_ values are visible. OFC assumes a tradeoff between the reliance on internal models and sensory feedback. Furthermore, the reliance on sensory feedback is upregulated when accurate internal models cannot be formed (e.g., due to uncertainty) (Franklin et al., 2012, 2017). We speculate that the shift in start and end points increased the uncertainty about the environment. This uncertainty especially increased for the blocked group but not for the random group since the latter group already experienced higher uncertainty inherent in their practice schedule. Therefore, the blocked group could have increased their feedback gains following the target shift allowing for more vigorous corrective responses when encountering the force field (Crevecoeur et al., 2019). Collectively, the increased feedback gains and thus the more vigorous corrections during the ongoing movement in the blocked group would essentially cancel out the difference in the force field prediction (FFCF) yielding in a similar motor net output (PD_*max*_).

We saw a group difference for the shifted-origin trials in both short- and long-term tests but no group difference in the long-term tests for the interpolation target. Possibly, a retrieval effect may have played a role. This refers to the phenomenon that relearning of a force field occurs at a more rapid rate than initial learning and of overcoming a warmup-decrement (Krakauer, 2005; Krakauer and Shadmehr, 2006; Haith and Krakauer, 2014; Huberdeau et al., 2015a,b; Schmidt et al., 2019). Maybe the warm-up decrement was overcome through the FF trials during the long-term tests until the long-term shifted origin tests started. Then, the better transfer performance of the random group, which was found during the short-term tests, could emerge again.

### State-Space Models Provide Further Explanations for the Contextual-Interference Effect

In addition to the experimental testing of the CIE, we developed SSMs to explain potential superior performance effects of the random practice schedule by the two-rate characteristic of the learning process. Except for the practice start, the extrapolation test, and partially long-term retention, the SSM could reproduce the performance trends in the behavioral data. Therefore, its underlying processes provide explanations to some but not all behavioral findings.

#### Practice

In the blocked group (Figures 7A,B), both processes were active throughout practice. When the target changed, activity in the fast process always increased, and decreased in the slow process. During the periods when the blocked group approached the same target 200 times, the context did not change and the Gaussian trial-by-trial learning had the strongest effect on the recurrent context. Whenever the target changed, the responsible context for the new target became active. Since the new context has been solely learned by trial-by-trial generalization and became active for the first time then, this resulted in a lower adaptation for the new context than for the preceding at the time the new context became active. When the new context became active, fast process activity increased and contributed more to the overall adaptation than at the end of the preceding context. Every time a new context became active, the preceding one was increasingly forgotten. The observation that first the fast process and then the slow process lead to adaptation when the target changed can be related to the respective process characteristics (Huberdeau et al., 2015b).

The fast process is sensitive to reward and its activity level rises fast, whereas the slow process seems to be more error-driven and rises more slowly. Regarding the underlying physiological cause, the literature suggests that adaptation, in the beginning, is achieved by stiffening the arm either as a result of an impedance control strategy (Milner and Franklin, 2005; Heald et al., 2018) or to upregulate feedback gains (Crevecoeur and Scott, 2014; Crevecoeur et al., 2019) as the internal model was inaccurate for the new target (Franklin et al., 2012). When targets changed after every 200 trials, participants probably analogously first used these reactive responses as a result of a reward-based mechanism and then—on a slower timescale—adapted an internal model which was able to predict the force field (Franklin et al., 2012). Another possibility could be that the visible change of the target addressed the explicit component of the learning process, which has been shown to resemble the fast process (McDougle et al., 2015). Though, we did not control for explicit and implicit processes and thus this remains speculative.

In the random group (Figures 7C,D), overall performance was determined by activity of the fast process until approximately halfway through practice. Toward the end of practice, the slow process became very active, and the activity of the fast process decreased. Both slow and fast processes revealed fluctuations which resulted from the continuous context switches. Whenever the target changed, the responsible context became active. The time that passed until the target was reached again caused a decrease in activity of the corresponding context. However, since the time was short, the decrease was only small, which resulted in the fluctuations.

#### Retention

Participants with a random practice schedule showed better short-term retention than participants with a blocked practice schedule. There were no differences between the long-term retention performances. Schweighofer et al. (2011) explained, without a statistical test, the increased immediate retention performance of the random group with a more pronounced activation of the slow process during practice. This ultimately led to a higher level of the slow process at practice end when compared to the slow process level of the blocked group. They explained that the slow process, which started from a higher level at practice end and then decayed slowly in the random group, led to better retention. In our study, we also observed a higher level of the random group’s slow process, yet the difference was not significant with a medium effect. It must be noted that we did not test retention for each target individually. With respect to the forgetting-and-reconstruction hypothesis, it could be that in the blocked group the earlier a target was practiced the more it got forgotten. So, the earliest practiced targets got considerably forgotten. In contrast to the random group in which all targets were practiced in a block-randomized manner and no target got forgotten more than another. Possibly, this difference between the slow processes of the two groups yields different average retention values and thus is reflected in the worse retention for the blocked group.

Joiner and Smith (2008) showed the slow process to be the main contributor for the adaptation level during long-term retention (24 h), whereas the fast process does not contribute. This holds true for our modeling results as the adaptation level during long-term retention is only influenced by the slow process level. Also, our SSM is able to reflect the decrease of adaptation for both groups after 24 h. However, it fails to reproduce the experimental finding that the groups’ performances do not differ significantly. Possibly, it is not only the decay of the slow process that is responsible for the long-term retention performance. Other mechanisms may happen, like a fractional transition of the fast process into the slow process as suggested by Criscimagna-Hemminger and Shadmehr (2008) or model-free learning mechanisms occurring along with error-based learning (Huang et al., 2011). With regard to this, our experimental procedure and state-space modeling did not allow us to verify if the described phenomenon happened. Additionally, maybe the decrease in retention results from the interference of daily task reaching movements, like grabbing a cup of coffee in front of you, and thereby promoting wash-out, which we did not consider with the SSM approach. Thus, this question cannot be addressed here and remains speculative.

#### Transfer

To be applicable to adaptation to multiple targets, SSM must also include multiple contexts (Schweighofer et al., 2011; Tanaka et al., 2012; Albert and Shadmehr, 2018). However, new targets did not appear after adaptation in any of these studies. This contrasts with our study, as the interpolation, extrapolation, and shifted origin targets were not practiced. Hence, we used a simplified approach to let the SSMs account for the new targets (section “Materials and Methods”). Our SSMs reproduced the statistical results we found in our behavioral analyses results with little exceptions (Supplementary Data 3). Therefore, it seems that the Gaussian trial-by-trial generalization can account for transfer to new targets. The SSMs provide a possible explanation for the better short-term interpolation transfer performance of the random group in light of the forgetting-and-reconstruction hypothesis. Since the activity of the slow process for the four contexts increased much more uniformly in random than in blocked practice and resulted in a higher value for the interpolation target, this possibly explains the higher transfer for the interpolation target of the random group. The SSM fits showed minor transfer for the extrapolation target for both groups stemming from the fast process. The fast process does not consist of context-specific states, and so cannot revert from a high value to zero within a single trial. For the shifted origin targets, the context of the practice target with the same direction served as the context of the respective shifted-origin target. This is a simplification in the sense that we do not consider whether the context of the direction is embedded in an intrinsic, extrinsic, or a mixed coordinate system (Berniker et al., 2014). However, for the purposes of our study, this simplification seems valid as the SSM reproduced the behavioral data.

The SSM can also account for the fact that no significant differences were found for the long-term interpolation test. In the absence of error, i.e., during EC trials or breaks, the adaptation level decays exponentially (Orozco et al., 2021). Due to the exponential decay, the difference of the slow process between the two groups which was possibly responsible for the group difference in the short-term interpolation test also quickly became smaller. As a result, significant differences no longer occurred after 24 h. The model data are in line with the experimental findings of the long-term transfer test for the shifted origin targets, i.e., a superior performance of the random group. The SSM supports the explanation based on a possible retrieval effect and warmup-decrement (section “Random Practice Yields Better Short-Term Transfer but Not Necessarily Long-Term Transfer”). Every second block during long-term retention and tests, we used FF trials. With them, the SSMs accounted again for learning and the values of the processes increased.

Despite support in the literature (Smith et al., 2006), there is criticism that SSMs cannot validly account for the underlying mechanisms of all savings or retrieval phenomena in force field adaptation (Herzfeld et al., 2014; Krakauer et al., 2019). Based on SSMs, savings are explained with the higher onset value of the slow process after being re-exposed to the force field (Smith et al., 2006). However, savings are also found after a prolonged washout period during which the slow process diminishes almost to zero (Zarahn et al., 2008). A possible extension to SSMs is the use of variable error sensitivities (Zarahn et al., 2008; Herzfeld et al., 2014; Coltman et al., 2019) or different parallel states as supposed by Lee and Schweighofer (2009).

### Operationalization of the Contextual Interference in Force Field Adaptation

The size of the CIE seems to be dependent on the type of variation practiced (Magill and Hall, 1990). In this regard, a certain amount of challenge seems to be critical (Guadagnoli and Lee, 2004). This means that, up to a certain degree, the more difficult or dissimilar the tasks are, the better participants would benefit from an interleaved practice schedule. In this adaptation study, participants practiced reaching to different targets in a force field. Even though, there is fractional transfer of learning between neighboring targets (Donchin et al., 2003; Howard and Franklin, 2015; Rezazadeh and Berniker, 2019), we propose that reaching to different directions can be considered dissimilar in the context of an CIE as it requires different joint movements (Morasso, 1981) and muscle activations (Flanders, 1991; Karst and Hasan, 1991; Thoroughman and Shadmehr, 1999). Furthermore, transfer of learning to neighboring targets seems to decrease with increasing direction difference (Gandolfo et al., 1996). Taken together, reaching to different directions in the force field may constitute a sufficient interference and thus a challenge in the context of the CIE to provoke better retention and transfer for the interleaved group. Studies in a similar laboratory setting, where practicing one task variation can presumably transfer to the others, also showed a CIE: Schweighofer et al. (2011) found a CIE in grip force pattern, Chalavi et al. (2018) in a visuomotor task, Lelis-Torres et al. (2018) in a manual aiming task, and Thürer et al. (2019), where force field magnitudes varied. We therefore considered the different reaching directions to be dissimilar enough to see a CIE.

### Neuronal Mechanisms Related to the Contextual-Interference Effect and State-Space Model

Recent studies have started to address the question of the underlying neuronal mechanisms related to the decomposition of adaptation into two distinct processes (Kim et al., 2015a; Sarwary et al., 2018; Farrens and Sergi, 2019). Kim et al. (2015a) demonstrated in a visuomotor adaptation that slow formation of memory relates to activity in the inferior parietal cortex and anterior-medial part of the cerebellum; and fast formation to areas in the prefrontal and parietal lobes and the posterior part of the cerebellum. Studies of the CIE associated the improved retention and transfer performance of a random schedule to increased activity in the parietal lobe (Thürer et al., 2018) or the dorsolateral prefrontal cortex (Kantak et al., 2010), and showed increased activity with blocked practice in the motor cortex (Kantak et al., 2010). SSMs as applied in our study are descriptive models of behavior and so do not allow us to infer the underlying neural mechanisms (Krakauer et al., 2019). Especially, it is yet unresolved whether the processes can be associated with short- and long-term memory to fully support the forgetting-and-reconstruction hypothesis (Schweighofer et al., 2011). As the CIE is detectable in both motor adaptation and skill learning studies, and these two types of motor learning are likely to have overlapping neural circuitry (Krakauer et al., 2019), future studies may further investigate the CIE and the neural differences between blocked and random practice which lead to the different behavioral results.

### Limitations

There are a few limitations that need to be considered. Firstly, we used two approaches (fit over all trials vs. by target) to compare adaptation speed by comparing the PD_*max*_ progression, which both come with limitations. Comparing τ over all trials obscures the difference between practicing targets block-wise vs. in a row, yielding a τ for the random group around four times larger than τ for the blocked group. However, if τ is compared by target, the occurring transfer of learning between the different targets and the decay of learning of a target until it is reached again is obscured for the random group. Furthermore, let t be the n^th^ trial for target i. For the blocked group, t would be trial number (n+200+n+400+n+600+n)/4 = 300+n on average. For the random group, t would be between trial numbers 4n-3 and 4n. Thus, on average, t appears earlier in the random schedule than in the blocked for the first half of the trials, where adaptation progresses most (4n < 300+n; *n* = 1 ≤ n ≤ 99). The blocked group would therefore have more practice trials before t. Secondly, the interspersed FF trials during the long-term tests may blur the results of the CIE. The savings or retrieval effect likely plays a more dominant role than the CIE for our long-term tests. Future research may assess the CIE after a 24 h break without an FF trials effect to gain more insights into the CIE in motor adaptation because, in motor skill learning, an increased retention is also observed after 48−72 h (Wright and Kim, 2019). Thirdly, our SSMs did not account for biomechanical differences of the different reaching directions (Molier et al., 2011; Rand and Rentsch, 2017). Another limitation of our SSM is that it did not account for possible non-linear error sensitivity (Fine and Thoroughman, 2007; Wei and Körding, 2009) or context-dependent decay (Ingram et al., 2013).

## Conclusion

The study shows that the CIE, which has been primarily investigated in motor skill learning studies, can partially lead to better retention and spatial transfer in motor adaptation tasks. Studying the influence of different practice schedules on retention and transfer is of theoretical as well as of practical interest. The study of the CIE in motor adaptation helps to better understand the underlying processes, as skill learning and motor adaptation are likely to make use of some shared neural circuitry and the causes of the CIE are still inconclusive. The study of the effects of different practice schedules also aims at providing practitioners with the most efficient practice schedules which ultimately may help foster the learning and execution of motor skills.

## Data Availability Statement

The raw data supporting the conclusions of this article will be made available by the authors, without undue reservation.

## Ethics Statement

The studies involving human participants were reviewed and approved by the Ethics Committee of the Karlsruhe Institute of Technology. The participants provided their written informed consent to participate in this study.

## Author Contributions

MH, AF, PM, BT, and TS were involved in the design of the study, involved in the interpretation and discussion of the results, provided critical feedback, revised, and contributed to the final manuscript. MH and PM carried out all data collection. MH carried out the data analysis and took the lead in writing the manuscript. All authors contributed to the article and approved the submitted version.

## Conflict of Interest

The authors declare that the research was conducted in the absence of any commercial or financial relationships that could be construed as a potential conflict of interest.

## Publisher’s Note

All claims expressed in this article are solely those of the authors and do not necessarily represent those of their affiliated organizations, or those of the publisher, the editors and the reviewers. Any product that may be evaluated in this article, or claim that may be made by its manufacturer, is not guaranteed or endorsed by the publisher.
