# Supplementary material for: Random Practice Enhances Retention and Spatial Transfer in Force Field Adaptation
Source: Front Hum Neurosci. 2022 May 4;16:816197. doi: 10.3389/fnhum.2022.816197 (PMC9116228; doi:10.3389/fnhum.2022.816197)
Supplement: Supplementary file 1 [file Data_Sheet_1.pdf]

Supplementary Material

Supplemental Tables

Supplemental Table S1: detailed description of the target ordering during short- and long-term transfer (in parentheses). Numbers are as follows. Practice targets: 1: 1.30, 2: 12, 3: 9, and 4: 7.30 h, 5: interpolation, 6: extrapolation, 7-10: targets with shifted origin in the same order as the practice targets.

Participant	EC				EC (FF)				EC		EC (FF)		EC				EC (FF)			
**1**	1	2	3	4	1	2	3	4	5	6	5	6	7	8	9	10	7	8	9	10
**2**	1	2	3	4	1	2	3	4	6	5	6	5	7	8	9	10	7	8	9	10
**3**	4	3	2	1	4	3	2	1	5	6	5	6	10	9	8	7	10	9	8	7
**4**	4	3	2	1	4	3	2	1	6	5	6	5	10	9	8	7	10	9	8	7
**5**	2	3	4	1	2	3	4	1	5	6	5	6	8	9	10	7	8	9	10	7
**6**	2	3	4	1	2	3	4	1	6	5	6	5	8	9	10	7	8	9	10	7
**7**	1	4	3	2	1	4	3	2	5	6	5	6	7	10	9	8	7	10	9	8
**8**	1	4	3	2	1	4	3	2	6	5	6	5	7	10	9	8	7	10	9	8
**9**	3	4	1	2	3	4	1	2	5	6	5	6	9	10	7	8	9	10	7	8
**10**	3	4	1	2	3	4	1	2	6	5	6	5	9	10	7	8	9	10	7	8
**11**	2	1	4	3	2	1	4	3	5	6	5	6	8	7	10	9	8	7	10	9
**12**	2	1	4	3	2	1	4	3	6	5	6	5	8	7	10	9	8	7	10	9
**13**	4	1	2	3	4	1	2	3	5	6	5	6	10	7	8	9	10	7	8	9
**14**	4	1	2	3	4	1	2	3	6	5	6	5	10	7	8	9	10	7	8	9
**15**	3	2	1	4	3	2	1	4	5	6	5	6	9	8	7	10	9	8	7	10
**16**	3	2	1	4	3	2	1	4	6	5	6	5	9	8	7	10	9	8	7	10

Supplemental Table S2: results of the grid search with bootstrapping. Values in the *RMSE* and R^2 columns are mean and 97.5% confidence intervals

A_s	A_f	b_f, b_s	*RMSE*	R^2
∈]0,1[	∈]0,1[	∈]0,1[	4-1: 0.29 (0.11)	0.74 (0.14)
			1-4: 0.29 (0.10)	0.70 (0.29)
			4-4: 0.30 (0.13)	0.70 (0.18)
		∈]0,0.5[	4-1: 0.28 (0.11)	0.74 (0.14)
			1-4: 0.29 (0.10)	0.72 (0.23)
			4-4: 0.30 (0. 3)	0.70 (0.19)
	∈]0.5,1[	∈]0,1[	4-1: 0.28 (0.11)	0.74 (0.14)
			1-4: 0.29 (0.09)	0.73 (0.15)
			4-4: 0.30 (0.13)	0.70 (0.19)
		∈]0,0.5[	4-1: 0.28 (0.10)	0.75 (0.14)
			1-4: 0.29 (0.10)	0.74 (0.14)
			4-4: 0.30 (0.13)	0.70 (0.16)
	∈]0,0.9]	∈]0,1[	4-1: 0.29 (0.09)	0.73 (0.14)
			1-4: 0.30 (0.10)	0.72 (0.35)
			4-4: 0.30 (0.13)	0.70 (0.18)
		∈]0,0.5[	4-1: 0.29 (0.11)	0.73 (0.18)
			1-4: 0.29 (0.10)	0.73 (0.23)
			4-4: 0.30 (0.11)	0.70 (0.23)
∈]0.9,1 [	∈]0,0.9]	∈]0,1[	4-1: 0.29 (0.09)	0.72 (0.17)
			1-4: 0.29 (0.09)	0.73 (0.17)
			4-4: 0.31 (0.11)	0.70 (0.18)
		∈]0,0.5[	4-1: 0.30 (0.12)	0.72 (0.16)
			1-4: 0.29 (0.06)	0.73 (0.17)
			4-4: 0.30 (0.13)	0.70 (0.18)
	∈]0.5,0.9]	∈]0,1[	4-1: 0.29 (0.11)	0.73 (0.18)
			1-4: 0.29 (0.09)	0.73 (0.17)
			4-4: 0.30 (0.12)	0.70 (0.19)
		∈]0,0.5[	4-1: 0.29 (0.11)	0.73 (0.18)
			1-4: 0.28 (0.08)	0.73 (0.15)
			4-4: 0.30 (0.12)	0.71 (0.19)

Supplemental Data S3: Detailed analysis of the model data

1.1 Practice

According to our experimental data analysis, we conducted an ANOVA for the model data for the practice phase. It revealed significant time (*F(1,30)*=315.515, $p<0.001$, $\eta_p^2=0.913$), group (*F(1,30)*=15.685, $p<0.001$, $\eta_p^2=0.343$), and interaction effects (*F(1,30)*=35.541, $p<0.001$, $\eta_p^2=0.541$). *Post-hoc* t-tests revealed that both groups adapted to the force field (blocked: *t(15)*=-11.186, $p<0.001$, $|d|=2.796$; random: *t(15)*=-13.963, $p<0.001$, $|d|=3.491$). The groups differed at practice start (*t(30)*=-6.626, $p<0.001$, $|d|=2.419$), but not at practice end (*t(21.482)*=-1.371, $p=0.071$, $|d|=0.819$).

In summary, the model data reproduced the behavioral results, except for a significant group difference at practice start which was not present in the experimental data.

1.2 Retention

We conducted two ANOVAs, one regarding short-term retention ([Group: Blocked vs. Random, Time: Practice end, Short-term]) and the other regarding long-term retention ([Group: Blocked vs. Random, Time: Practice end, Long-term]). The ANOVA for the short-term retention revealed significant group (*F(1,30)*=32.960, $p<0.001$, $\eta_p^2=0.988$), time (*F(1,30)*=46.655, $p<0.001$, $\eta_p^2=0.609$), and interaction effects (*F(1,30)*=12.191, $p=0.002$, $\eta_p^2=0.289$). *Post-hoc* t-tests found a superior performance for the random group against the block group only in short term retention (*t(30)*=6.416, $p<0.001$, $|d|=2.343$). Both groups showed a decrease with time (blocked: *t(15)*=6.675, $p<0.002$, $|d|=1.669$; random: *t(15)*=2.632, $p=0.019$, $|d|=0.658$).

The ANOVA for the long-term retention revealed a significant time effect (*F(1,30)*=351.523, $p<0.001$, $\eta_p^2=0.921$) but no significant interaction effect (*F(1,30)*=2.617, $p=0.116$, $\eta_p^2=0.080$), which resembles the experimental findings. Unlike the experimental findings, we found a significant group effect (*F(1,30)*=24.628, $p<0.001$, $\eta_p^2=0.541$). *Post-hoc* t-tests revealed a significant decrease in performance during the long-term retention for both groups (blocked: $t(15)=13.493$, $p<0.001$, $|d|=3.373$; random: $t(15)=13.057$, $p<0.001$, $|d|=3.264$), alike the behavioral results. In contrast to our behavioral findings, we found a significant difference in the long-term retention test between the groups ($t(30)=6.416$, $p<0.001$, $|d|=2.343$).

In summary, the SSM reproduced the short-term retention performance of the experimental data, showing a better retention for the random group. Consistent with the behavioral results, the SSM was able to replicate the decline in performance in the long-term retention test for both groups. However, the model data revealed a significant group difference in the long-term retention test that was not present in the experimental data.

1.3 Spatial transfer

Analogously to our experimental statistics, we tested the model data for the interpolation, extrapolation, and shifted origin targets for both short- and long-term transfer. First, we tested for short-term transfer. The one-sample t-tests vs. 0 revealed transfer for all short-term transfer tasks for both the blocked (interpolation: *t(15)*=12.910, $p<0.001$, $|d|=3.228$; extrapolation: *t(15)*=2.437, $p<0.001$, $|d|=0.609$; shifted origin: *t(15)*=12.068, $p<0.001$, $|d|=3.017$) and the random group (interpolation: *t(15)*=22.432, $p<0.001$, $|d|=5.608$; extrapolation: *t(15)*=4.518, $p<0.001$, $|d|=1.129$; shifted origin: $t(15)=16.218$, $p<0.001$, $|d|=4.054$). Then, we tested for each target which group performed better. The t-tests showed a better performance for the interpolation and extrapolation target for the random group (interpolation: *t(30)*=5.502, $p<0.001$, $|d|=2.009$; extrapolation: *t(30)*=2.217, $p=0.035$, $|d|=0.847$). Further, the modelled performance was better for the random group for the shifted origin targets (*t(30)*=4.605, $p<0.001$, $|d|=1.681$).

We tested long-term transfers analogously to the short-term transfers. The one-sample t-tests vs. 0 showed transfer for both the blocked and the random group for the interpolation target (blocked: *t(15)*=4.606, $p<0.001$, $|d|=1.151$; random: *t(15)=5.488*, $p<0.001$, $|d|=1.372$). However, the tests showed transfer to the extrapolation target only for the random group, but not for the blocked group (blocked: *t(15)*=0.009, $p=0.993$, $|d|=0.002$; random: *t(15)*=-3.216, $p=0.006$, $|d|=0.804$).Both groups showed transfer to the shifted origin targets (blocked: *t(15)*=20.986, $p<0.001$, $|d|=5.246$; random: *t(15)*=31.579, $p<0.001$, $|d|=7.895$).

Then, we tested for each target if the groups' performances differ to each other. The t-tests showed no differences for the interpolation ($t(30)$=0.248, p=0.806, |d|=0.090) and extrapolation target ($t(30)$=-1.940, p=0.062, |d|=-0.708). The t-test to compare the shifted origin targets for the long-term transfer revealed a better transfer for the random compared to the blocked group ($t(30)$=2.323, p=0.027, |d|=0.848). Except for the found transfer for the extrapolation target in the random group, these findings resemble those of the behavioral data.

To sum up, the SSM reproduced the behavioral results of all transfer tests, except for the extrapolation target. For the latter, the modelled data showed a low transfer for both groups during short-term tests and for the random group during long-term tests, though the behavioral data showed no transfer.
